# Keyword Index for Volume 98

**DOI:** 10.1038/sj.bjc.6604447

**Published:** 2008-06-10

11q13 950

^131^I-MIBG 1053

^18^FDG 1597

25-OCH_3_-PPD 792

5-Aza-2′deoxycytidine 1810

5-fluorouracil 716

8-hydroxydeoxyguanosine 580

ABCG2 1515

adaptation 25

ADCC 1217

adenocarcinoma *in situ* 165

adherens junctions 256

adiponectin 370

adjuvant therapy 25, 1301

adolescence 1475

advanced gastric cancer 316, 1398

advanced non-small cell lung cancer 1769

advanced NSCLC 558

Affymetrix U133 Plus 2.0 array 1403

African 992

age 328

age distributions 277

age-dependent prevalence 646

ageing 619, 677

AIF 803

Akt 803, 1094, 1348, 1525

AKT1 E17K 1533

AKT/mTOR pathway 931

alcohol 1582, 1857

allele frequency 1125

ALT 1467

alternative splicing 129

amino acid transporter 742

anaemia 323

anal cancer 1264

androgen receptor 1094

angiogenesis 250, 776, 923, 1250, 1312, 1348, 1366, 1619, 1646, 1731

annexin 426

anorexia 300

anthracycline-pretreated 1500

anthropometrics 1288

anti-angiogenesis 1250

antibiotics 161

anti-tumour immunity 1258

AP-1 915

Aplidin 1966

apoptosis 370, 399, 803, 824, 941, 1109, 1803

archival formalin-fixed paraffin-embedded tissue 1403

aromatase inhibitors 1494

array CGH 434

Asian and Western populations 9

aspirin 989

asynchronous contralateral breast cancer 728

ATP-binding cassette transporters 380

atypical hyperplasia 45

autologous vaccine 1336

autophagy 399

Axl tyrosine kinase receptor 1141

AZD3409 1951 

*β*-catenin 1886

*β*-catenin-independent signalling 1437

Barrett's metaplasia 1985

basement membrane 974

BCP ALL 1125

BCRP 857

berberine 363

bevacizumab 1366

bexarotene 1380

BIBW 2992 80

biliary stricture 1548

biliary tract cancer 1548, 1675

biological effect study 1951

biological predictive factors 143

biomarkers 587, 776, 1366, 1731

biopsy 1182

birth outcome 183

birth weight 179, 660, 664, 1288

bisphosphonates 1736

bladder cancer 466

bleomycin 388

BMI-1 1662

body mass index 194, 1582

bone metastasis 809, 1736, 1753

bone turnover 1753

BORIS 571

bortezomib 1500

brachytherapy 450

brain tumour 1285

*BRCA1* 1457

*BRCA1* and *BRCA2* 502

BRCA1/2 2006

*BRCA2* 1457

breast cancer 9, 25, 39, 210, 289, 370, 564, 571, 636, 641, 734, 784, 840, 870, 989, 992, 1132, 1141, 1147, 1327, 1380, 1485, 1494, 1500, 1525, 1536, 1641, 1741, 1753, 1790, 1870, 1876, 1182, 2006

breast cancer mortality 206

breast neoplasm 217

breast screening 1741, 1759

breast-conserving surgery 289

British black women 277

burnout 1046

bystander effect 1244

cachexia 300

calcium 1574

cancer biomarker 981

cancer growth 1250

cancer mortality 1012

cancer patients 443

cancer risk 660

cancer risk model 1457

cancer site 1012

cancer stem cells 1389, 1670, 1886

cancer suppressor gene 410

cancer therapy 517

capecitabine 309, 316, 1204, 1320

carbonic anhydrase inhibitor 129

carbonic anhydrase IX 129

carboplatin 1774

carcinogen exposure 619

carcinogenesis 245, 965, 1653

Carcinoid 1053

carcinoma 627, 981

carcinoma of the oral cavity 1039

carcinoma of unknown primary 1425

cardiovascular disease 1586

case-control comparison 1125

case-control studies 161, 168, 174, 179, 206, 652, 992, 1570, 1586, 1852

*CASP8* 1434

caveolin-1 931

CD133 1389

CD24 756

CD44 756

*CDKN2A* 434

CDKN2A (p16) 1264

CEA 328, 1217

CEC 1731

cell cycle 523, 824

cell death 98

cell proliferation 981

central laboratory 907

centrosome 345

ceramides 98

ceramidoids 98

cervical cancer 15, 863, 1292

cervical screening 1704

cervical smear 1292

cetuximab (Erbitux®, C225) 120

checkpoint 523

CHEK2*1100delC mutation 728

chemokines 1682

chemoprevention 232, 1157

chemoradiotherapy (CRT) 1670

chemoresistance 335, 803, 1085

chemosensitisation 792

chemotherapy 22, 86, 309, 542, 693, 728, 734, 870, 1197, 1210, 1327, 1769

childhood cancer 1285, 1876

childhood leukaemia 225, 1006

Chk1 523

cholangiocarcinoma 309, 418, 1675

chordoma 434

chromatin 1467

chromosomal radiosensitivity 1723

chromosome instability 345

CIMP 1555

circadian rhythm 1753

circulating progenitor cells 776

cirrhosis 1166

cisplatin 86, 803, 881, 1085, 1803

cJun 915

clinical response 77

clinical study 1312

clinical trial 1452

clinicopathological features 277

c-MYC 1985

cohort 1001, 1929

cohort study 183, 194, 529, 1864

colon cancer 587, 875, 1312, 1366, 1619

colorectal cancer 60, 71, 323, 328, 335, 668, 716, 997, 1034, 1217, 1434, 1555, 1614, 1682, 1810, 1824, 1886, 1999

colorectal carcinogenesis 1437

colorectal neoplasms 98

combination therapy 1234

combined modality 1210

communication 1508

concurrent chemoradiotherapy 881

constitutional chromosome deletions 1929

consultation 1508

contact-inhibition 256

continuity of care 529

contrast agent 1784

cortactin 950, 956

cost-effectiveness 1166, 1762

cost-utility 1166

counter-matching 45

COX-2 1102

CpG arrays 466

CPT-11 335

CT scan 875

CTCF 571

CUP 1425

CXCR4 1682

cyclin D1 950

cyclopentenyl cytosine 1226

CYFRA 21-1 77

CYP1B1 564

CYP3A 91

CYP3A4 1797

*CYP3A4* transgenic 1630

cytochrome *c* release 1525

cytochromes *P*450 564, 1797

cytology 154

cytoskeleton 256

dairy protein 1574

data modelling and analysis 1023

database 816

decision aid 1769

decision-making 1830

decompensated cirrhosis 1161

dendritic cells 106, 784

depression 1934

deprivation 1006

detection 263

diabetes 1582

diagnosis 60, 323, 1425, 1540, 1562

diagnostic validity 1934

dietary iron 194

dietary lignans 636

differentiation 1415

dihydropyrimidine dehydrogenase 832

dipeptidyl aminopeptidase IV 818

disease/relapse-free survival 1641

DKK1 1147

DNA adducts 1959

DNA damage 480

DNA methylation 816, 824, 1881

DNA methyltransferase 1716

DNA repair 1614

DNA replication 981

docetaxel 542, 558, 894, 1305, 1500, 1608, 1710, 1774

domestic fuel 1586

dominant-negative mutant 915

doxorubicin 1515

drug metabolism 91

drug resistance 564, 923, 1068

drug therapy 98

drug-drug interaction 1320

ductal carcinoma *in situ* (DCIS) 137

duration of chemotherapy 1197

dysplasia 457

dyspnoea 294 

early life exposure 1288

early stage 1191

Ebp1 1132

E-cadherin 489

EDD 1085

education 1012

effectiveness 641

EGF receptor 956

EGFR 154, 256, 450, 923, 1076

eIF2*α* 443

elderly patients 558

electrochemotherapy 388

electroporation 388

EMA 1562

*EML4-ALK* 1536

employment 708, 1342

endocrine sensitivity 22

endometrial adenocarcinoma 45

endometrial cancer 1076, 1288, 1582

endometrial carcinoma 1662

endometrial neoplasms 194

endometrioid ovarian cancer 282

endothelium 1731

England 633

EphB4 845

ephrinB2 845

EPIC 1574

epidemiology 161, 183, 641, 652, 1285, 1582, 1852, 1870

epidermal growth factor receptor 80, 418, 749, 832, 1118

epidermal growth factor receptor (*EGFR*) mutation 907

epigenetics 496, 1485, 1515, 1881

epirubicin 720

epithelial ovarian cancer 1191

epithelial-mesenchymal transition 489, 1993

Epo binding 1059

ER negative 1380

ERBB2 1525

*ERCC1* 1398

ERK1/2 1525

erlotinib 1774

erythropoietin receptor 1059

ethnicity 633

etoposide 693

Europe 1012

everolimus 923

evidence 1944

excision repair cross- complementing gene 1 832

expression 1059

extracolorectal cancer 997

extremity soft tissue sarcoma 1403

Ezrin 256 

FADD 950

FAK 1274

familial cancer 1876

familial risk 840, 997

family history 199

Fanconi anaemia 1653

Fanconi genes 1452

FDG PET 875

fibroblast 1886

fine-needle aspiration cytology 818

first-line chemotherapy 720

FISH 154

FOLFOX 1398

follow up 875

formalin-fixed paraffin- embedded; gene expression; prognostic assay 1999

FOXO1 1068

Foxp3 148, 1258

free radicals 580

FRNK 1274

funding 1944

fusion transcript 1536 

G2 523

gallbladder carcinoma 309

gastric cancer 54, 148, 542, 824, 832, 965, 1301, 1443

gastrin 1696

gastrointestinal 1696

gastrointestinal cancer 1536

gastrointestinal stromal tumours 684, 1762

gastro-oesophageal cancer 888

gastro-oesophageal junction tumours 965

GD2 263

Gd-DTPA 1784

gefitinib 71, 716, 907

gemcitabine 558, 881, 1226, 1327, 1710, 1916

gene chip 363

gene expression 245, 587, 1327, 1845

gene expression profiling 1023, 1264, 1403, 1425

gene signature 1662

general population 708

genetic alteration 749

genetic predisposition 728, 1443, 1723

genetic testing 1457

genistein 1485

genomic imbalances 434

genomics 756

geography 1944

geriatric oncology 517

germline mutation 474

GGH 1555

ghrelin 300

GHRH 1790

ginsenoside 792

Gli-1 1670

glioblastoma 113, 1431

glioma 652, 1830

glutathione-*S*-transferase 2006

grapefruit 1630

green tea 168

growth arrest-specific gene 6 1141

growth factors 250

GSTP1 polymorphism 2006

guidelines 1944

gynaecological 1774 

haem iron 194

haematology 1046

HDAC 604

head and neck cancer 1274, 1723

head and neck squamous cell carcinoma 619, 956

health education 1176

Hector Battifora mesothelial antigen-1 818

Hedgehog (Hh) 1670

*Helicobacter pylori* 689

hepatic transporters 91

hepatitis C virus 580

hepatocellular carcinoma 1161, 1166

HER1/EGFR 1774

HER2 1525

HER2/neu 1076

herceptin 1525

hereditary syndrome 1876

HGF receptor 1102

HIF-2*α* 965

high dimensionality 1023

high-field MR 1784

histone deacetylase inhibitors (HDACIs) 1234

histopathology 45

*HLA-DPB1* 1125

HNSCC 950

Hodgkin's disease 183

hormone receptors 1662

hormone replacement therapy 194

hormone therapy 870

hormone-refractory prostate cancer 1094

hospital referral 1176

HPV 627

HPV screening 15

HPV typing 1704

HPV vaccination 1704

HPV vaccines 15

hTERT 1467

hTR 1467

human epidermal growth factor receptor 2 418

human papillomavirus 646, 863, 1264

human parvovirus B19 611

humanised monoclonal antibody 1525

hypermethylation 1690, 1716

hypoxia 129, 965, 1975

hypoxia-inducible factor-1 (HIF-1) 1975 

ICAM2 1357

ifosfamide 720

imatinib 1633, 1762

imatinib mesylate 684

immunocytochemistry 263

immunohistochemistry 137, 418, 426, 450, 580, 587, 604, 1662

immunotherapy 784, 1336

*in vivo* tumour inhibition 1250

incidence 165, 633, 989

indisulam 1320

inequality 668

infant acute leukaemia 664

infectious aetiology hypothesis 225

inflammation 91, 1443, 1646

informed consent 1769

INI1 474

interaction analysis 1508

internal ribosome entry 1696

interstitial pneumonitis 1870

intra-arterial chemotherapy 1039

intracranial invasion 363

intrauterine factors 664

invasion 113, 356, 1274

invasive breast cancer 277

invasive ductal carcinoma 1993

invasiveness 1348

ionising radiation 1226

Iran 1857

irinotecan 693, 1210, 1312, 1619

iron 580

job satisfaction 1046

job stress 1046 

ketoconazole 1797

Ki-67 742, 1525

kinesin spindle protein 894

kinetic analysis 1597

*KIT* 684 

lactate 1975

lactation 189, 992

laminin-332 974

lapatinib 1076

large G protein 410

larynx 950

LAT1 742

late effect 1001

lay terms 34

leukaemia 168, 1006, 1633

linear and nonlinear clearance 900

linear discriminant analysis 457

lipocalin 1540

liposarcoma 1467

LMO4 537

locally advanced 1210

locally advanced breast cancer 1745

LOH 1830

lomeguatrib 1614

long-term follow-up 863

long-term risk 870

long-term survival 720

longitudinal studies 1342, 1475

loss of heterozygosity 410, 1690

lower urinary tract symptoms 1903

lung adenocarcinoma 1710

lung and prostate cancer 1790

lung cancer 232, 270, 1109, 1602, 1608, 1653

lymph node metastasis 1389, 1682, 1824

lymphocyte 1845

lymphoma 161 

macrophage 1118

MAD 1985

male radiographer 1759

malignancy 91

malignant glioma 345

malignant melanoma 106

mammary stem cell 1485

mammographic density 210

mammography 217, 641, 1182

markers 1830

markov models 1762

mass screening 217

mathematical model 113

matrix metalloproteinases 250, 766

matuzumab 900

MCAK 1824

MCM 1548

MCM7 1264

Mdm2 (Hdm2) 4

Mdm4 (MdmX, Hdm4, HdmX) 4

mechanisms of genomic alterations 1653

medical record linkage 1570

MEFs 480

melanoma 179, 1922

men 1012, 1864

meningioma 652

menopause 1288

menstrual cycle phase 39

Merlin 256

mesothelioma 1562

Met 1102

meta-analysis 547, 1934

metabolic syndrome 194

metabolism 1630

metalloproteinases 689, 1820

metastasis 86, 106, 931, 1415

metastatic 1500

metastatic breast cancer 1916

metastatic gastric carcinoma 1305

methylation 410, 466, 496, 1147, 1431, 1810

methyl-CpG-binding domain proteins 1881

metronomic chemotherapy 1312, 1619

microarray analysis 480

microarrays 1023, 1264, 1327, 1425

microenvironment 250, 809

microRNA 410

microsatellite 619

mistletoe lectin 106

mitochondrion 1839

mitotic kinesin 894

*MLL* rearrangements 664

MMP-2 1274

MMP-8 766

MMTV-erbB2 1380

mobile phone 652

modelling studies 270, 1166

modifiers 2006

molecular targeting 941

monoclonal antibody 749, 900

morphological types 199

mortality 1864

mouse brain 1784

MRI 113, 289

mRNA 1431, 1662

MS-275 1234

mTOR 923

MUC-1 784, 1562

mucin glycoprotein 1675

multicentre prospective phase II 907

multidrug resistance 857, 1515

multimodality 734

multiple myeloma 1966

murine models 1966

muscle atrophy 443

mutation 154, 619, 1533

mutational analysis 684

MV3 106

myosin IIA 596 

natural products 792

near-infrared Raman spectroscopy 457

neoadjuvant chemotherapy 289, 734, 1327

neoplasms 294, 1586

NER 120

nested case-control study 1295

neuroblastoma 263

neuroendocrine 1053

neurotoxicity 1959

NF-*κ*B 71, 335

NGAL 1540

nimotuzumab 749

nitric oxide 1803

nitric oxide synthase 1839

NKD1 1437

NM23-H1 363

NO-aspirin 1157

nodal dissection 54

noncoding RNA 981

non-Hodgkin 161

non-small cell lung cancer 77, 154, 742, 749, 907, 915, 1118, 1536, 1716

non-steroidal anti-inflammatory drugs 232, 1781

normal tissue response 1845

NOS 1803

NPC 363

nuclear factor-*κ*B 611

nuclear survivin 1109

nude mice 1525

nutrition 300 

*O*^6^-methylguanine-DNA methyltransferase 1614

odds ratio 1295

oesophageal adenocarcinoma 1102, 1985

oesophageal cancer 547, 689, 974, 1670, 1857

oestrogen 766

oestrogen receptor 370, 571, 636, 766

older patient 517

oncogene 1533

oncology 1046, 1508, 1597

opium 1857

optical diagnosis 457

oral cancer 633

oral contraceptives 1781

oral squamous cell carcinoma 1258, 1357

oropharynx 627

OSI-420 1630

OSI-774 1630

osteoclast 809

osteoclastogenesis 809

osteolysis 809

outcome 537, 1029

outcome prediction 1830

ovarian cancers 282, 489, 720, 845, 1068, 1085, 1197, 1415, 1452, 1781, 1803, 1910

overall survival 489, 1641

oxaliplatin 120, 309, 542, 1034, 1204, 1959

oxidative stress 580, 1157

oxygen 294

p53 4, 1431, 1803

p70^S6k^ 443

PA2G4 1132

paclitaxel 316, 1068

paediatric brain tumour 474

paediatrics 1570

PAK1 1132

palliative care 294

*Panax notoginseng* 792

pancreatic cancer 537, 881, 1226, 1389, 1540, 1548, 1690

pancreatic neoplasms 189

PanIN 1540

papillary thyroid carcinoma 611

paracrine 245

parathyroid hormone 1753

parenteral oestrogen 697

parity 199, 1295, 1781

participating patients 34

*PDGFRA* 684

pegylated liposomal doxorubicin 1916

penetrance 474

peptide-binding pockets 1125

peroxisome proliferating- activated receptor 1415

PET 1597

pH control 129

pharmacodynamics 1633, 1959

pharmacoepidemiology 232

pharmacogenetics 1633

pharmacogenomics 1710

pharmacokinetics 80, 98, 517, 1320, 1633, 1797, 1959

phase I study 80, 894, 1029, 1320

phase I/II study 1034, 1305

phase II study 716, 1336

*Phellinus linteus* 1348

phosphoinositide-dependent protein kinase 1 1415

phosphorylation 1132

physical activity 174, 1864

PI3K pathway 974

PKB 1525

PKR 443

platinum 1959

platinum resistance 1452

policy 641

poliglumex 1608

polymorphisms 689, 857, 1434, 1443

polyoxomolybdate 399

pooled analyses 282

population density 225, 1006

population mixing 1006

population movements 225

population pharmacokinetic 900

population-attributable fraction 199

population-based 179

postoperative adjuvant chemotherapy 596

PR1A3 1217

predicting factor 1389

pregnancy 183, 189

prematurity 1570

prenatal exposure 660

prenyl transferase inhibitor 1951

pre-operative radiotherapy 450

preoperative therapy 1745

prevention 1292, 1380

primary health care 323, 1176

principal components analysis 457

progesterone receptor 282, 571, 636

progesterone receptor B 1141

PROGINS 282

prognosis 148, 418, 426, 537, 627, 931, 956, 1102, 1258, 1716, 1820, 1824, 1876, 1910

prognostic factors 742, 832, 845, 974, 1029, 1039, 1161, 1745, 1993

prognostic markers 604, 1403

progress 1191

proliferation 113, 370, 1109, 1244

promoter methylation 1555

prospective 1574

prospective studies 39, 1342

prostate 245, 1852

prostate cancer (PCa) 22, 250, 356, 502, 604, 697, 756, 792, 1234, 1244, 1574, 1903

prostate pathology 502

prostate-specific antigen 1176

proteasome inhibitor 1500

protein 1059

proteomics 426

pyruvate 1975

pyruvate dehydrogenase kinase 1 (PDK-1) 1975 

qPCR 587

QRT-PCR (quantitative real-time polymerase chain reaction) 1845

quality of life 25, 54, 888, 1494, 1903

quality-adjusted survival 25

quantitation 1597

quantitative reverse-transcriptase polymerase chain reaction (qPCR) 1641 

radiation 1210, 1852

radiation therapy 728, 1039, 1845

radiation-induced bystander effects 1839

radiochemotherapy 1204

radiographer gender 1759

radionuclide 1053

radioresistance 1357

radiosensitisation 749, 792, 1226

radiosensitivity 345

radiotherapy 734, 870, 1001

rapamycin 931

reactive oxygen spices (ROS) 1068

real-time PCR 1999

receptor tyrosine kinase 1525

rectal cancer 143, 450, 1204, 1210

recurrence 1085, 1922

red meat 194

redox 1157

refractory 716

regulatory T cells 148

re-induction 22

relative deprivation 633

relative risk 199

relative survival 328

renal cell carcinoma 496, 931, 941, 1336

replication 120

reproductive history 189, 992

resection 113

resistance 1525

responder to uracil-tegafur 596

response to therapy 1525

retinoic acid metabolism-blocking agents (RAMBAs) 1234

return-to-work 1342

rexinoid 1380

rhabdoid tumour predisposition syndrome 474

risk 636, 863, 1434, 1929

risk assessment 840

risk factors 168, 210

risk prediction 270

RRM1 1710

RRM2 1710

RT-PCR 1830

*RUNX3* 1690 

S-1 1034, 1301, 1305

sarcoma 1633

SB-715992 894

screening 206, 641, 646, 1182, 1602

screening history 1292

SDF-1*α* 1682

second malignancies 870, 1001

secreted phospholipase A_2_ 587

semaxinib 1619

seminal vesicle 356

senescence 245, 250, 1244

sensitivity 1602, 1934

sentinel lymph node 1922

serous 1085

service delivery 529

SFRP 1147

side population 380

signal transduction inhibitors 1633

signalling 370

signalling pathway 1839

single-step selection 1515

siRNA 1357

Smac 941

small molecule inhibitors 4

small-cell lung cancer 693

SMF 210

smoking 1475, 1582

Snai1 489

Snai2 480

SNIP 1641

SNPs (single nucleotide polymorphisms) 282, 1820, 1845

socio-economic factors 217

socioeconomic status 277, 668

South Africa 1586

SOX 1034

SOX2 824

soy 9, 1485

SPARC 1810

specificity 1602

splice variant 1250

squamous cell carcinoma 380, 766, 1274

stage 668, 888

stage I lung adenocarcinoma 596

staging 328, 547

stem cells 380, 480, 660, 857

stomach 457

stomach cancer 1295

stomach cancer survivor 708

stroma 1886, 1993

sunitinib 1762

superoxide dismutase 776

supertypes 1125

supportive care need 1903

surgery 1745

surgical resection 537

surveillance 1166

survival 39, 263, 1029, 1741, 1820

survival analysis 217, 1118

survival prediction 77

survivin 345, 627, 1109

survivin-deltaEx3 1109

syndecan-1 1993

systematic review 60, 697

systemic therapy 1769 

tamoxifen intolerance 1494

Tarceva 1630

targeted therapy 677, 684, 857

telomerase 1467

telomeres 677

temozolomide 1614

temsirolimus 1797

testicular cancer 174

testicular neoplasms 171

tetrathiomolybdate 776

therapeutic response 537

therapy 1161

three drug combination 693

thrombospondin-1 1312

thymidylate synthase 143

thyroid nodule 818

thyroid peroxidase 818

timing of surgery 39

tissue microarray 137, 426, 956

TNF 1443

tobacco 1586, 1653, 1857

tongue 766

topoisomerase-II*α* 1910

transcript 1059

transforming growth factor-*β*_1_ 356

transitional cell carcinoma 86

transitions in care 529

translation 1696

trastuzumab 1525

treatment 888, 1944

treatment decisions 1769

treatment delay 1197

treatment induced bone loss 1736

T_reg_ 1258

trial 54

trial results 34

triple negative 277

triplets 720

*TS* 1398

tumor invasion 1646

tumour advancement 845

tumour classification 137, 1425

tumour development 1525

tumour load 1922

tumour marker 1675

tumour stem cells 756

tumour suppressors 1485

tumours 1059, 1784

twins 171, 1475

tyrosine kinase inhibitor 80 

ultrasound 1285

uptake 1759

urbanisation 1006

urokinase-type plasminogen activator 356

USPIO 1784

uterine cancer 45 

vaccination 646

vascular disruption 388

vascular endothelial growth factor 418

vascular invasion 931

vasculature 1731

VEGF 450, 1250, 1366, 1784

VEGF_165_b 1366

VEGF-C 1389

vimentin 596

viral protein 611

virus DNA 611

VN/66-1 1234 

Wnt 1147

Wnt signalling 1437

women 1012

work ability 708, 1342

wound healing 1646 

XIAP 941 

young adults 1475 

zoledronic acid 1753

